# Folate receptor-α targeted near-infrared fluorescence imaging in high-risk endometrial cancer patients: a tissue microarray and clinical feasibility study

**DOI:** 10.18632/oncotarget.23155

**Published:** 2017-12-11

**Authors:** Leonora S.F. Boogerd, Charlotte E.S. Hoogstins, Katja N. Gaarenstroom, Cornelis D. de Kroon, Jogchum J. Beltman, Tjalling Bosse, Ellen Stelloo, Jaap Vuyk, Philip S. Low, Jacobus Burggraaf, Alexander L. Vahrmeijer

**Affiliations:** ^1^ Department of Surgery, Leiden University Medical Center, Leiden, The Netherlands; ^2^ Department of Gynecology, Leiden University Medical Center, Leiden, The Netherlands; ^3^ Department of Pathology, Leiden University Medical Center, Leiden, The Netherlands; ^4^ Department of Anesthesiology, Leiden University Medical Center, Leiden, The Netherlands; ^5^ Department of Chemistry and Center for Drug Discovery, Purdue University, West Lafayette, IN, USA; ^6^ Centre for Human Drug Research, Leiden, The Netherlands; ^7^ Leiden Academic Center for Drug Research, Leiden, The Netherlands

**Keywords:** targeting, biomarker, tumor-specific imaging, FRα

## Abstract

**Objective:**

Detection and resection of all malignant lesions is pivotal in staging and cytoreductive surgery (CRS) of endometrial cancer (EC). Intraoperative EC detection could be enhanced using OTL-38, a fluorescent-labelled folate receptor-α (FRα) targeted imaging agent. The objectives of this study were to investigate which subgroups of high-risk EC patients express FRα and assess feasibility of intraoperative EC detection using OTL-38.

**Results:**

FRα expression on TMA was significantly correlated with tumor type (*p* < 0.01). Eighty-two percent of serous and clear cell carcinomas showed FRα expression. Four patients were enrolled in the clinical study. Using fluorescence imaging all omental (*n* = 3) and lymph node (LN) metastases (*n* = 16) could be clearly identified, including one otherwise undetected omental metastasis. However, false-positive fluorescence was identified in 17/50 non-metastatic LNs, caused by OTL-38 targeting of FRβ, expressed by tumor-associated activated macrophages.

**Conclusions:**

This study describes high FRα expression in serous and clear cell EC and demonstrates the first experience of intraoperative FRα-targeted tumor detection in patients with these subtypes of EC. Although all metastases could be clearly identified using OTL-38, the role of tumor-associated macrophages should be further evaluated.

**Methods:**

Immunohistochemical (IHC) staining of FRα expression was performed on tissue micro arrays (TMA) of 116 patients with high-risk EC features. Patients with either serous or clear cell EC, planned for staging or CRS, were eligible for inclusion in the clinical study and received an intravenous dose of 0.0125 mg/kg OTL-38, 2-3 hours prior to surgery. Resected lesions, identified by standard-of-care and/or fluorescence imaging, were histopathologically assessed for FRα and tumor status.

## INTRODUCTION

Endometrial cancer (EC) can be categorized in type 1 and type 2, based on etiology and clinicopathologic features [1]. Type 1 EC are commonly estrogen-dependent, low-grade, endometrioid adenocarcinomas, accounting for 80% of all EC. Type 2 EC account for the remaining 20% and represent a more aggressive, high-grade tumor type with a poorer prognosis [2, 3]. These tumors are generally non-endometrioid, i.e. serous or clear cell, and unrelated to estrogen exposure. High-risk EC is defined as a combination of several factors including non-endometrioid EC, more advanced disease stages/age, high-grade and lymphovascular space invasion [4]. Although EC is conventionally categorized in type I and II, improved understanding of the molecular landscape of EC has resulted in subdivision of EC in four molecular subtypes with improved prognostic significance, among others p53 mutant EC [5]. Each type and risk group of EC requires a different surgical approach because of distinctive tumor characteristics. For type I, low-grade EC the extent of pelvic organ resection and lymphadenectomy depends on the tumor stage, whereas a complete staging is recommended for clinically early stage serous and clear cell carcinomas, due to higher rates of metastatic disease [6]. A complete surgical staging includes a total hysterectomy with bilateral salpingo-oophorectomy (BSO), pelvic and para-aortic lymph node sampling, omentectomy, and several peritoneal biopsies of predefined spots [7]. Depending on the presence of metastases during surgical staging, patients will either be monitored via follow-up or treated with adjuvant therapy [1].

Both during staging and cytoreductive surgery (CRS) of EC it is of utmost importance to identify tumor lesions with high accuracy. Surgeons are dependent on inspection and palpation (in case of open surgery) for intraoperative distinction between tumor and normal tissue. Histopathological analysis on frozen specimens obtained during the procedure can provide additional information, but is only performed on clinically suspicious lesions. Furthermore, non-suspect, but malignant lesions can easily be missed when visual inspection and palpation are the sole means for identification. An innovative technique that can assist in real-time intraoperative tumor detection is near-infrared (NIR) fluorescence imaging [8]. This technique is based on administration of a fluorescent agent and real-time detection of fluorescence using a dedicated NIR imaging system. A new era in the field of NIR fluorescence imaging has emerged with the clinical testing of tumor-targeted fluorescent contrast agents [9]. These agents consist of a tumor-targeting ligand, e.g. a peptide, antibody, nanobody etc., conjugated to a NIR fluorescent dye. One of the most promising agents currently available for clinical testing is OTL-38, a NIR fluorescent-labelled peptide targeting the Folate Receptor-α (FRα) [10]. The FRα, an isoform of the folate receptor, is anchored on the cell membrane and binds folic acid with high affinity. Expression of FRα on normal tissue is restricted to the apical surface of few epithelial tissues. However, marked overexpression in several tumor types, including EC, makes the FRα an attractive candidate for targeted imaging and therapy [11, 12].

Safety and feasibility of FRα-targeted tumor detection using OTL-38 have been demonstrated in ovarian and lung cancer [10, 13]. Application of OTL-38 in EC surgery could be especially valuable in high-risk EC patients, who have a high likelihood of extra-uterine disease [7]. In these high-risk EC patients, real-time fluorescence guidance with OTL-38 during staging and CRS may provide enhanced visualization and detection of more metastatic lesions. The aim of this study was to assess which high-risk EC patients could benefit from FRα-targeted imaging. Although general overexpression of FRα in EC was previously described [12, 14], specific FRα expression in only high-risk EC patients, including both non-endometrioid and endometrioid EC, has not been demonstrated. Therefore, expression of FRα was assessed on a tissue micro array (TMA), consisting of 116 tissues derived from EC patients with high-risk features. Based on these results, feasibility of NIR fluorescence intraoperative tumor detection using OTL-38 was studied in patients with serous or clear cell EC, scheduled for staging or CRS.

## RESULTS

### TMA-study

**Figure 1:**
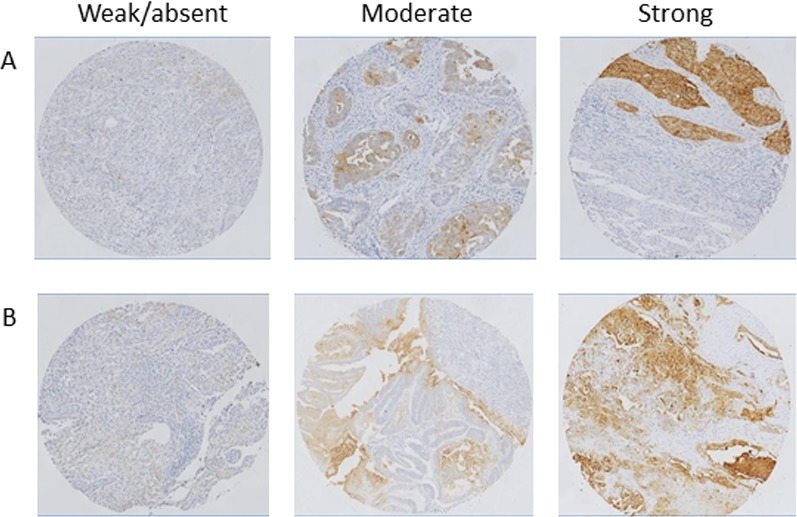
Representative examples of FRα expression status in endometrioid and clear cell EC Shown are tissue cores with respectively weak, moderate and strong intensity of FRα staining in endometrioid (**A**) and clear cell carcinoma (**B**). Magnification: 10×.

Tissue cores of 101 patients were suitable for assessment of FRα expression. Clinicopathological characteristics and expression scores are shown in Table 1. Figure 1 shows representative examples of weak, moderate and strong intensity of FRα expression, in both endometrioid and non-endometrioid cancer. FRα expression was found in 63% of endometrioid cancers and in 82% of non-endometrioid cancers. Strong FRα expression was found in 38% of all endometrioid cancers compared to 46% of non-endometrioid cancers. A significant correlation (*p* < 0.01) between the pattern of FRα expression, i.e. homogenous vs. heterogenous, and tumor type was found. The majority of serous endometrial carcinomas (73%) showed homogenous FRα expression, while clear cell carcinomas showed a more heterogenous FRα expression pattern (65%). Endometrioid cancers showed both homogenous and heterogenous FRα expression in respectively 33% and 30% of all scored cases. Furthermore, a significant association between p53 status, i.e. wildtype or mutant, and FRα expression was found (*p* = 0.01). All (11/11) homogenous FRα-expressing non-endometrioid cancers showed a mutant p53 status.

**Table 1:** Clinicopathologic patient characteristics in relation to FRα expression

	Total, *n* (%)	FRα expression in tumor cells, *n* (%)			
	(*n* = 101)	absent	heterogeneous	homogenous	*P* value
**Age**					
<60	33 (33%)	15 (45%)	13 (39%)	5 (15%)	0.057
60–70	26 (26%)	6 (30%)	11 (42%)	9 (35%)	
>70	42 (42%)	11 (26%)	12 (29%)	19 (45%)	
**FIGO stage 2009**					
I	36 (36%)	15 (42%)	9 (25%)	12 (33%)	0.355
II	20 (20%)	5 (25%)	10 (50%)	5 (25%)	
III	35 (35%)	9 (26%)	13 (37%)	13 (37%)	
IV	9 (%)	3 (33%)	4 (44%)	2 (22%)	
**Tumor type**					
endometrioid	73 (72%)	27 (37%)	24 (33%)	22 (30%)	0.004
serous	11 (11%)	2 (18%)	1 (9%)	8 (73%)	
clear cell	17 (17%)	3 (18%)	11 (65%)	3 (18%)	
**Grade**					
1	11 (11%)	3 (27%)	5 (45%)	3 (27%)	0.082
2	5 (5%)	0 (0%)	1 (20%)	4 (80%)	
3	85 (84%)	29 (34%)	30 (35%)	26 (31%)	
**Depth of myometrial invasion**					
<50%	19 (20%)	4 (21%)	6 (32%)	9 (47%)	0.255
>50%	76 (80%)	28 (37%)	26 (34%)	22 (29%)	
**Lymphovascular space invasion**					
Absent	34 (40%)	15 (44%)	7 (21%)	12 (35%)	0.367
Present	48 (57%)	15 (31%)	19 (40%)	14 (29%)	
unknown	2 (2%)	1 (50%)	1 (50%)	0 (0%)	
p53 status, endometrioid cancer					
Wildtype	56 (77%)	21 (38%)	20 (36%)	15 (27%)	0.472
Mutant	17 (23%)	6 (35%)	4 (24%)	7 (41%)	
**p53 status, non-endometrioid cancer**					
Wildtype	9 (32%)	3 (33%)	6 (67%)	0 (0%)	0.013
Mutant	19 (68%)	2 (11%)	6 (32%)	11 (100%)	

Abbreviations: A description of the different staining patterns, i.e. absent, heterogenous and homogenous, is provided in the method section.

### Clinical study

#### Patient characteristics

Four patients with EC (serous carcinoma; *n* = 3 and clear cell carcinoma; *n* = 1) were included in the clinical study (Table 2). Three patients underwent a staging procedure and one patient CRS. The patient undergoing CRS (patient #4) was diagnosed with a large mass in the fundus uteri (5.5 cm) and two suspect peritoneal depositions in the omentum (respectively 1.3 cm and 2.8 cm) on preoperative computed tomography imaging. CRS consisted of uterus extirpation, BSO and omentectomy.

**Table 2:** Patient and tumor characteristics of patients included in the clinical study

Patient No.	Age	Type of Surgery	Diagnosis	Primary tumor *in situ*	Suspicion metastatic disease
1	53	Staging/ open surgery	Serous adenocarcinoma	Yes	Yes, enlarged suspect LNs
2	68	Staging/ laparosopic	Serous adenocarcinoma	Yes	No
3	68	Staging/ laparoscopic	Serous adenocarcinoma	No	No
4	76	Cytoreduction/ open surgery	Clear cell carcinoma	Yes	Yes, two omental lesions

Abbreviations: LNs = lymph nodes.

### Safety and pharmacokinetics

All patients received 0.0125 mg/kg OTL-38 over 1 hour and no infusion was stopped or intermitted. Two patients experienced possibly related AEs: one patient experienced a mild muscle spasm and another patient mild pruritus. Both AEs were self-limiting. No clinically relevant changes in blood pressure or pulse rate were observed. The maximal concentration for each dose was obtained directly after the end of the infusion. The elimination half-life was approximately 86 min.

### Intraoperative fluorescence imaging

**Figure 2:**
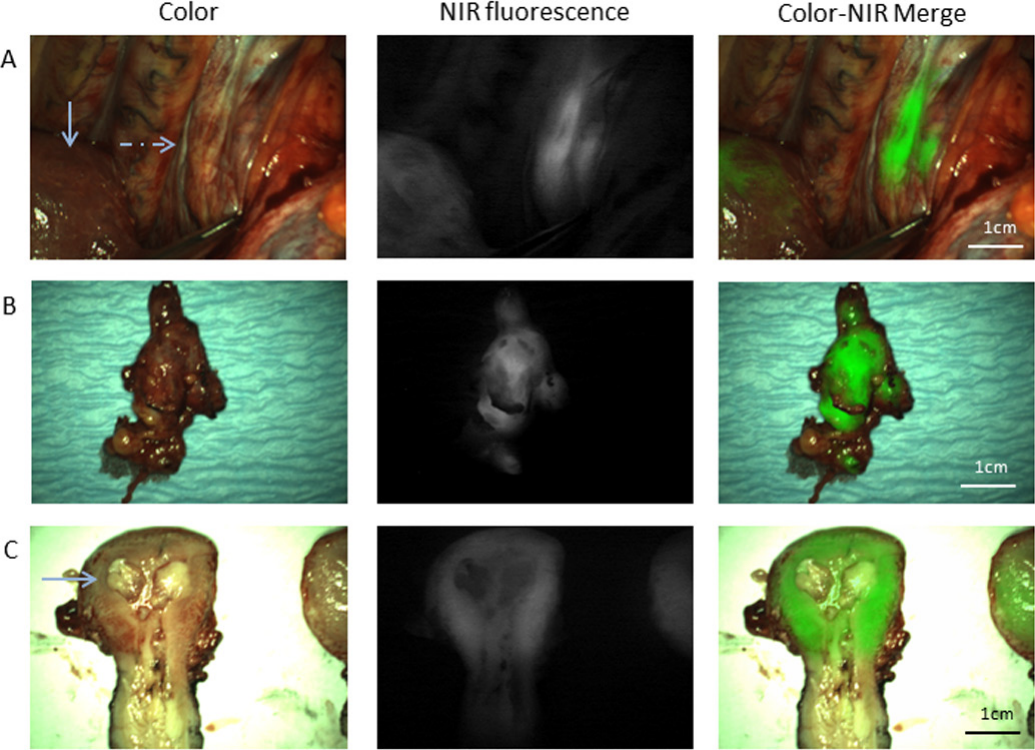
Fluorescence imaging of a primary uterine serous adenocarcinoma and of the metastatic lymph nodes (**A**) Intraoperative identification of para-aortic, metastatic lymph nodes (dashed arrow), located beneath a layer of overlying tissue (patient #1). The normal arrow indicates the fluorescence signal arising from the uterus. (**B**) *Ex vivo* fluorescence imaging of the resected para-aortal lymph nodes (patient #1), that show a clear fluorescence signal. (**C**) *Ex vivo* fluorescence imaging of the bisected uterus (patient #1). The fluorescence signal, detected during surgery, appears to be mainly arising from normal adjacent background uterine tissue instead of the primary tumor.

Intraoperative fluorescence imaging allowed clear detection of tumor lesions using an exposure time of less than 60 ms. During surgery a fluorescent signal arising from the uterus could be detected in all patients with the primary tumor still *in situ*, with a mean tumor-to-background ratio (TBR) of 6.4 (SD = 4.7; range: 2.9–13, Table 3). However, after slicing of the resected specimen, adjacent uterine tissue without tumor (#1 and #2) appeared more fluorescent than the signal arising from the tumor (Figure 2). In patient #4, almost no normal uterine tissue was present because of the bulky size of the tumor. The intraoperative fluorescence signal therefore probably originated from the primary tumor, instead of from normal uterine tissue.

**Table 3:** Relation between fluorescence imaging and final histopathology

Patient No.	Primary tumor		Lymph nodes		Omentum		Other biopsies	
	PA	Fluorescent (TBR)	PA	Fluorescent (TBR)	PA	Fluorescent (TBR)	PA	Fluorescent (TBR)
1	Malignant	Yes^*^ (3.2)	Malignant (16/22)^**^	Yes, all (6.3)	Benign	No	Benign	No
2	Malignant	Yes^*^ (13)	Benign	Yes, some (3.0)	Benign	No	Benign	No
3	n/a	n/a	Benign	Yes, some (1.8)	Benign	No	Benign	No
4	Malignant	Yes^*^ (2.9)	n/a	n/a	Malignant^***^	Yes, all (2.4)	n/a	n/a

Abbreviations: n/a = not applicable; TBR = tumor-to-background, shown as mean; ^*^a fluorescence signal arising from the uterus was seen during surgery ^**^in 16/22 lymph nodes macrometastases were found; ^***^two omental depositions were identified on preoperative imaging, but during surgery a third lesion was identified solely by NIR fluorescence imaging. Final histopathological analysis showed tumor cells in all three omental lesions.

In two of four patients (#1 and #4) metastases were found in respectively 16/22 LNs and in three omental lesions. In the remaining two patients, no metastases were found during staging procedures. All histologically proven metastatic malignant lesions (19/19) could be identified by fluorescence imaging, with a mean TBR of 6.3 (SD = 4.5; range 3.2–14.1) in metastatic LNs (16/19) and a mean TBR of 2.3 (SD = 0.2; range 2.1–2.5) in omental metastases (3/19). Importantly, one omental lesion was not seen during visual inspection and only identified by fluorescence imaging. Histopathological analysis of this lesion revealed a small deposit of clear cell carcinoma. Fluorescence imaging enabled clear detection of all histologically proven metastatic LNs, even when these were located beneath a layer of peritoneum or other overlying tissue (Figure 2).

Furthermore, a total of 50 LNs were resected that did not contain tumor cells. Seventeen out of these 50 LNs were detected by fluorescence imaging (patient #1, #2 and #3). Mean TBR of these false-positive LNs was 2.5 (SD = 1.3; range 1.5–6.2). No other false-positive lesions were identified, i.e. no fluorescence was detected in biopsies of the bladder, the diagraphm and recto-uterine (Douglas) pouch. Sensitivity, specificity and positive predictive value of fluorescence detection of LNs was 100% (16/16), 70% (39/56) and 48% (16/33) respectively.

### Histopathology

**Figure 3:**
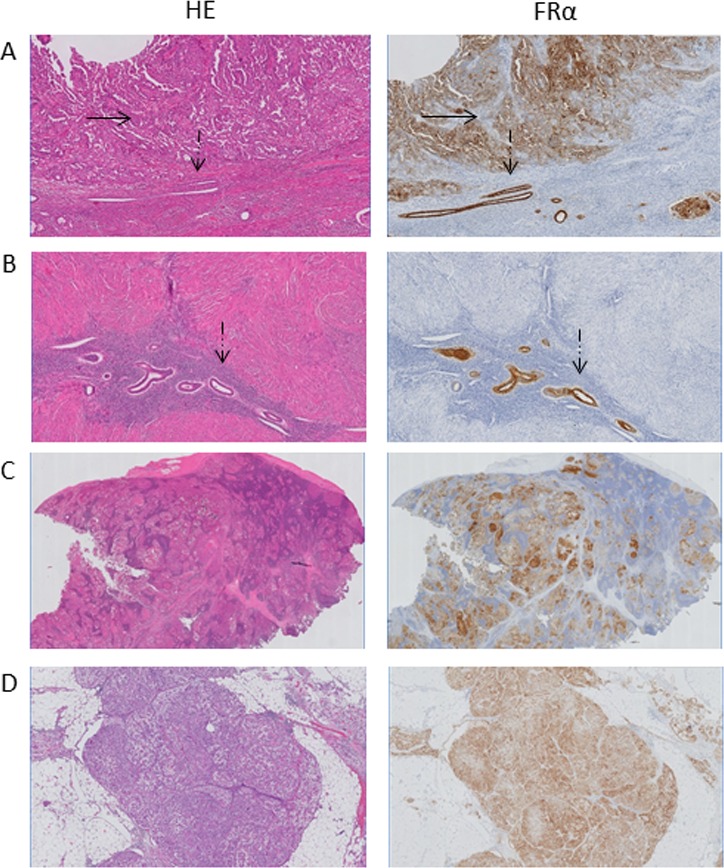
Histopathological evaluation of resected lesions (**A**) Immunohistochemical staining for FRα of a uterine tumor shows FRα expression in cancer cells (black arrow, patient #1) and in normal epithelial cells (dashed arrow). Magnification: 5×. (**B**) Immunohistochemical staining for FRα of non-malignant adenomyosis tissue of the uterus (patient #2). Magnification: 5×. (**C**) Immunohistochemical staining for FRα of a clinically suspect and fluorescent lymph node shows positive FRα expression in the lymph node follicels (patient #1). The FRα expression correlates with the presence of tumor cells. Magnification: 0.5×. (**D**) Immunohistochemical staining for FRα of a fluorescent omental lesion, that contained tumor cells, shows positive FRα expression (patient #4). Magnification: 2×.

Histopathological analysis of EC lesions showed a circumferential staining pattern of FRα in malignant cells of both clear cell and serous cancer origin (Figure 3). FRα expression was also found in adjacent normal uterine epithelial cells and in adenomyosis tissue (patient #2, Figure 2), possibly explaining the weakened fluorescence intensity of uterine tumors compared to adjacent ‘normal’ uterine tissue.

**Figure 4:**
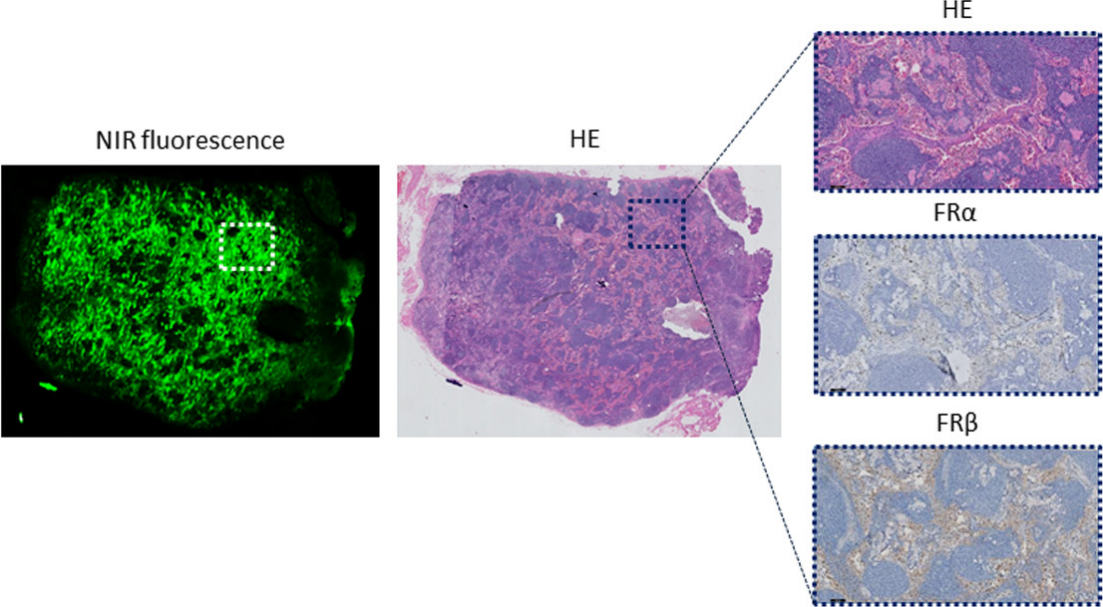
Histopathological evaluation of a false-positive lymph node Shown are a NIR fluorescence image, haematoxylin & eosin (HE) staining, and FRα and FRβ staining of a (fluorescent) LN that did not contain tumor cells. Fluorescence is mainly seen in the sinuses and not in the follicles. The magnified images (HE, FRα and FRβ) show the lack of FRα staining, while the sinuses show a positive FRβ staining. Magnifications: 1× and 10×.

In all metastatic LNs, an intense FRα expression was seen in lymph node follicles, while expression in sinuses was weak (Figure 3). In contrast, FRα expression was absent in all (fluorescent) false-positive LNs. Additional staining experiments however showed FRβ expression in the sinuses of these LNs which explained the false-positive fluorescence signal. The FRβ is expressed by activated macrophages and is also targeted by OTL-38 (Figure 4).

## DISCUSSION

Prior to the clinical study, we first performed a TMA study with tissue of EC patients with high-risk clinical features to select those EC patients who may benefit most from FRα-targeted tumor imaging [4]. Optimally, selection of EC patients with homogenous FRα expression would result in targeting of all FRα-expressing tumor cells and consequently, low chances to miss tumor lesions in the clinical setting. A significant association between FRα expression and tumor type was shown, with positive expression in the majority of non-endometrioid cancers (82%). Remarkably, 73% of serous type EC showed homogenous FRα expression, which makes particularly this group of patients suitable for FRα imaging. Although a substantial part of high-risk EC patients (63%) also showed FRα expression, we chose to include only non-endometrioid cancer patients in this clinical proof-of-concept study. Non-endometrioid EC is by definition high-grade and all these patients require staging or CRS. Several other studies support our findings and show upregulation of FRα in non-endometrioid EC and a correlation with tumor grade [12, 14, 15]. Limitations of the TMA part of this study are inherent to IHC analysis, such as variation in staining techniques, scoring systems and quality of antibodies. Notwithstanding, results of the current study corroborated results of the TMA study by Brown Jones *et al.* [14], who also showed a significant association between FRα overexpression and high-risk EC. Our study differs because FRα expression was evaluated among a subset of EC patients with high-risk features. Additionally, this is the first study to show a significant association between homogenous FRα expression and a mutant p53 status in non-endometrioid EC. Routinely performed evaluation of the p53 status may therefore assist in patient selection for FRα-targeted agents. We did not perform a FRβ staining on the TMA, because previous literature describes that FRβ is more prevalent on stromal cells than on cancer cells [16, 17]. Yet, a systematic comparison between FRα and FRβ expression on endometrial cancer tissues with enough stromal tissue may be interesting.

The most important prognostic factor for EC survival is the presence of LN metastases. Therefore, pelvic and para-aortic lymph node sampling is recommended as an integral part of a surgical staging procedure for a subset of EC patients with a high likelihood of metastatic disease, such as non-endometrioid EC patients [6]. The goal is to identify patients with nodal disease who will most likely benefit from adjuvant therapy [18]. The morbidity and mortality, costs and importantly, impact on patient survival associated with this procedure are however all subject to debate, especially in early stage EC patients [19]. In an attempt to better match the extent of the surgical staging procedure with the risk of LN metastasis, a surgical algorithm has recently been adopted for early stage EC patients [20]. Another method that has been advocated as alternative staging procedure is sentinel lymph node (SLN) mapping. Recently published results of a large multicenter SLN study suggest high accuracy of SLN detection in 340 early EC patients using indocyanine green (ICG) as fluorescent tracer [21]. Cervical injection of ICG led to successful mapping of at least one SLN in 86% of patients and nodal metastases were correctly identified in 35 of 36 patients (97%). ICG is a safe and relatively inexpensive fluorescent dye, that has been extensively studied for SLN mapping in multiple tumor types, such as vulvar and cervical cancer [22, 23]. Although ICG proved its suitability as lymphatic tracer, it is not tumor-specific and does not bind to tumor cells. The use of ICG during gynecologic cancer surgery is therefore limited to the detection of SLNs. OTL-38 is an example of a tumor-targeted fluorescent tracer, that can selectively highlight tumor cells that express FRα [10]. OTL-38 has therefore the potential to aid gynecologists in real-time detection of distant metastases, i.e. peritoneal metastases, as well as more reliable removal of metastatic pelvic and para-aortic LNs.

In the current study, all metastatic LNs were detected using fluorescence imaging with OTL-38. Importantly, LNs located below a layer of ± 1 cm of tissue, such as para-aortic LNs, could be clearly identified. Nevertheless, three out of four patients showed false-positive LNs during staging procedures. The fluorescence signal arising in those LNs appeared related to expression of FRβ, which is also targeted by OTL-38 as shown by histopathology evaluation. FRβ is selectively expressed on activated macrophages and is therefore explored as imaging target to detect lesions of inflammatory conditions [24]. Recently the role of FRβ in tumor tissues has been elucidated showing FRβ expression in tumor-associated macrophages [25]. In a study of thousand tumor sections, Shen et al. showed that FRβ is mainly expressed on tumor-associated macrophages in stromal cells [17]. Importantly, the percentage of positively stained cells correlated with tumor stage and LN involvement, suggesting that expression of FRβ might be an indication of the metastatic potential of a tumor. Further research is needed to clarify whether the apparently non-malignant LNs identified with fluorescence imaging are involved in premetastatic niche formation. If so, resection of these LNs may yield clinical benefit. Thus, despite the fact that all metastatic LNs expressed FRα, the widespread implementation of OTL-38 for metastatic LN detection remains limited until the role of FRβ is elucidated.

Although no clear intraoperative distinction between uterine tumor and background tissue could be made using OTL-38, this is not relevant in staging or CRS of EC because all patients undergo a total hysterectomy. Previous studies report on constitutive FRα expression on normal uterine epithelium, but high expression on various EC tumor tissues [12, 26]. An additional finding observed after histopathological analysis of uterine tissues was strong FRα expression in adenomyosis cells, which has been previously described in 17/18 endometriosis samples [27]. Since the aim of surgery in severe endometriosis patients is to resect all visible lesions, it is plausible that OTL-38 may enhance intraoperative detection in those patients, enabling better patient outcome.

In conclusion, this study demonstrates the first application of OTL-38 for intraoperative tumor detection during staging and cytoreductive surgery in patients with either serous or clear cell EC. Prior to the clinical study, a TMA study on tissues from high-risk EC patients demonstrated a significant association between FRα expression and tumor type. In the clinical study, all malignant LNs and omental metastases could be clearly identified using OTL-38. However, until the role of FRβ in false-positive LNs is unambiguously established, the added value of OTL-38 for detection of metastatic LNs is limited.

## MATERIALS AND METHODS

### Tissue selection

High-risk (stage IB-III) EC samples were collected from collaborating institutions within the *Trans*PORTEC consortium, as previously described [4]. High-risk EC was defined using inclusion criteria for the PORTEC3 study [28]. Specimen of 116 patients were included in this tissue micro array (TMA) study, and tissue microarrays contained 1-mm tumor and tumor/stroma cores in triplicate. Clinicopathological characteristics, including tumor type, stage, grade and p53 status of all cases have been described previously [4].

### Immunohistochemical staining and evaluation

Immunohistochemistry was performed as described previously using the monoclonal antibody (Mab) 26B3.F2 (certified Folate Receptor alpha IHC Assay Kit, Biocare Medical) [29]. The Mab26B3.F2 is highly specific for FRα without cross-reactivity to the other FR, e.g. FRβ, FRγ or FRδ. For validation of the staining protocol lung adenocarcinoma was used as positive control and normal liver as negative control.

Blinded, independent evaluation of IHC staining was performed by two observers (L.B. and C.H.). Discrepancies were resolved by reviewing the relevant scores with a board certified pathologist (T.B.). A tumor core was rejected and not included in the analysis if it was missing or if >75% of the core was insufficient for evaluation. Staining was scored as absent/weak (0), moderate (1+) or strong (2+). A positive FRα expression was defined when >5% of tumor cells showed a moderate or strong FRα expression. A core was considered negative when none or <5% of tumor cells showed FRα expression. The overall intensity of staining of a case was recorded for the intensity that was seen in the majority of cores. Homogeneity was defined when all three cores showed similar intensity of FRα expression.

### Clinical study

This trial was approved by the Medical Ethics Committee of the Leiden University Medical Center and was performed in accordance with the ethical standards of the Helsinki Declaration. Four patients with a high suspicion of primary serous or clear cell carcinoma, planned for either staging or CRS by laparotomy or laparoscopy, were included. Main exclusion criteria were pregnancy, history of anaphylactic reactions and impaired renal function (defined by eGFR < 50 ml/min/1.73 m^2^) or liver function (defined as values greater than 3× the upper limit of normal (ULN) for ALT, AST, or total bilirubin).

### Tracer administration and surgical procedure

Patients received a 1 hour intravenous infusion of 0.0125 mg/kg OTL-38, 2-3 hours before the start of surgery. The investigational product, OTL-38, has been extensively described [10]. Tolerability assessments (blood pressure, pulse, peripheral oxygen saturation, respiratory rate, ECG, temperature and skin assessments) and blood collection for pharmacokinetics and routine laboratory tests were performed regularly from just before administration up to 24 hours post-dosing. Adverse events (AEs) and use of concomitant medication were recorded. All surgical procedures were performed by an experienced gynecological oncologist. First the surgical field was searched for metastases by usual visual and tactile methods (the latter only in case of open surgery). Thereafter, the open or laparoscopic Artemis imaging system was used to identify NIR fluorescent lesions as described previously [30]. All tumor tissue identified, irrespective of the method, was resected if this was surgically feasible and clinically considered important by the operating gynecological oncologist. Each resected lesion was marked as fluorescent or non-fluorescent and as clinically suspected or not suspected for malignancy.

### Analysis

All resected lesions were routinely examined by an experienced pathologist for tumor status. A fluorescent tumor positive lesion was considered a true positive lesion, a fluorescent tumor negative lesion a false positive lesion and a non-fluorescent tumor positive lesion a false negative lesion. Additionally, immunohistochemical (IHC) staining for FRα was performed. IHC staining of FRβ was performed to evaluate the cause of false positive fluorescence. Placenta was used as positive control for FRβ staining. The negative control was assessed by using the secondary antibody only, without the primary antibody.

### Statistical and image analysis

Statistical analysis was performed using the IBM SPSS for Windows 20.0 software. Correlation of patient and tumor characteristics, including p53-status, with FRα expression was assessed with *t*-test for continuous variables and with χ^2^ test of Fisher’s exact test for categorical variables. In all statistical tests, a *p*-value of <0.05 was considered statistically significant.
